# Impact of cryoprotective agents on human gut microbes and in vitro stabilized artificial gut microbiota communities

**DOI:** 10.1111/1751-7915.14509

**Published:** 2024-06-15

**Authors:** Giulia Alessandri, Sonia Mirjam Rizzo, Leonardo Mancabelli, Federico Fontana, Giulia Longhi, Francesca Turroni, Douwe van Sinderen, Marco Ventura

**Affiliations:** ^1^ Laboratory of Probiogenomics, Department of Chemistry, Life Sciences, and Environmental Sustainability University of Parma Parma Italy; ^2^ Department of Medicine and Surgery University of Parma Parma Italy; ^3^ Microbiome Research Hub University of Parma Parma Italy; ^4^ APC Microbiome Institute and School of Microbiology, Bioscience Institute National University of Ireland Cork Ireland

## Abstract

The availability of microbial biobanks for the storage of individual gut microbiota members or their derived and artificially assembled consortia has become fundamental for in vitro investigation of the molecular mechanisms behind microbe–microbe and/or microbe–host interactions. However, to preserve bacterial viability, adequate storage and processing technologies are required. In this study, the effects on cell viability of seven different combinations of cryoprotective agents were evaluated by flow cytometry for 53 bacterial species representing key members of the human gut microbiota after one and 3 months of cryopreservation at −80°C. The obtained results highlighted that no universal cryoprotectant was identified capable of guaranteeing effective recovery of intact cells after cryopreservation for all tested bacteria. However, the presence of inulin or skimmed milk provided high levels of viability protection during cryoexposure. These results were further corroborated by cryopreserving 10 artificial gut microbiota produced through in vitro continuous fermentation system technology. Indeed, in this case, the inclusion of inulin or skimmed milk resulted in a high recovery of viable cells, while also allowing consistent and reliable preservation of the artificial gut microbiota biodiversity. Overall, these results suggest that, although the efficacy of various cryoprotective agents is species‐specific, some cryoprotectants based on glycerol and the addition of inulin or skimmed milk are preferable to retain viability and biodiversity for both single bacterial species and artificial gut microbiota.

## Graphical abstract

Evaluation of the ability of different cryoprotective buffers to preserve the viability of several bacterial species representative of the human gut microbiota as well as the viability and biodiversity of artificial gut microbiota obtained through an in vitro continuous fermentation model system after cryopreservation at −80°C.

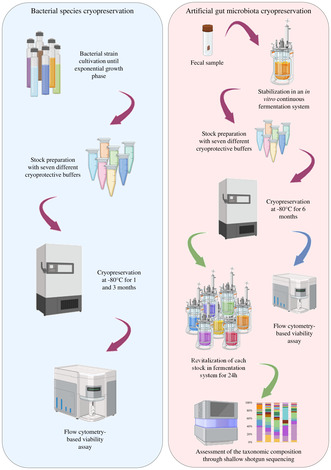

## INTRODUCTION

The human gut microbiota is a complex, dynamic and diverse ecosystem that plays a crucial role in influencing host health status (Fan & Pedersen, 2021; Milani et al., 2017; Sommer et al., 2017). For this reason, in recent decades, the scientific community has made substantial efforts to perform large‐scale assessments of this intricate microbial consortium using high‐throughput, culture‐independent approaches (Arumugam et al., 2011; Hu et al., 2013; Milani et al., 2016; Nagata et al., 2022; Wallen et al., 2022). These efforts have provided an impressive general overview of the bacterial composition and deduced functional features that characterize the human gut microbiota, also highlighting how this microbial ecosystem is modulated by various parameters such as diet, geographical origin, age, diseases or drug and/or prebiotic/probiotic intake (Claesson et al., 2012; Qin et al., 2022; Tarracchini et al., 2021; Vich Vila et al., 2020; Wirbel et al., 2019). However, these techniques exclusively provide predictive and descriptive information, preventing an in‐depth insight into the molecular mechanisms that underpin microbe–microbe, microbe–host and microbe–dietary compound interactions (Amrane et al., 2018; Wang et al., 2015). In this context, human gut microbiota fermentation models have recently been proposed as highly informative in vitro/ex vivo approaches to investigate microbe–microbe interplays as well as the impact that biotic and abiotic factors may exert on the intestinal microbial ecosystem, avoiding the ethical concerns associated with in vivo studies (Guzman‐Rodriguez et al., 2018; Isenring et al., 2023; Li et al., 2019; Mabwi et al., 2021; Nissen et al., 2020; Ryan et al., 2021; Singh et al., 2022). Among the various fermentation systems, continuous fermentation models have attracted scientific interest since they better mimic the intestinal environment when compared to *in batch* cultivation, guaranteeing strict control of various parameters, in particular pH, as well as nutrient input and exhausted growth medium removal to avoid the accumulation of toxic metabolites (Guzman‐Rodriguez et al., 2018; Isenring et al., 2023; Payne et al., 2012; Sardelli et al., 2021). Furthermore, these models allow extended cultivation times, and have been claimed to generate the establishment of a so‐called “artificial gut microbiota”, i.e., an in vitro stabilized bacterial community that, although characterized by a reduced biodiversity when compared to a donor's faecal inoculum, constitutes a valid representation of the more complex intestinal microbiota (Bircher, Geirnaert, et al., 2018; Bircher, Schwab, et al., 2018; Isenring et al., 2023). Indeed, the availability of artificial gut microbiota offers substantial advantages as they can be used multiple times without depending on the continuous recruitment of volunteers. Furthermore, they allow a wide range of taxonomic combinations to always be available in the laboratory, as well as higher reproducibility of the experiments since artificial gut microbiota are not subject to the variations that, instead, occur over time in the microbial community of a donor (Bircher, Geirnaert, et al., 2018; Bircher, Schwab, et al., 2018; Silverman et al., 2018). However, the long‐term storage of the artificial gut microbiota is an essential prerequisite to ensure the viability of the incorporated species and, therefore, to maintain its desired and predetermined bacterial biodiversity (Biclot et al., 2022; Bircher, Geirnaert, et al., 2018; Bircher, Schwab, et al., 2018; Chen, Hu, et al., 2022; Chen, Huo, et al., 2022; Chen, Li, et al., 2022; Smirnova et al., 2019).

Cryopreservation at temperatures ranging from −80°C to −196°C is the most widely employed method for long‐term bacterial storage (Barzegari et al., 2014; Bircher, Geirnaert, et al., 2018; Bircher, Schwab, et al., 2018; Smirnova et al., 2019). However, ice formation may cause lethal damage to bacterial cells, making the use of cryoprotective agents necessary (Bircher, Geirnaert, et al., 2018; Bircher, Schwab, et al., 2018; Smirnova et al., 2019). In this context, the effects of various cryoprotectants on bacterial cell viability and physiology during freezing, storage and thawing have been reported for pure cultures or faecal samples (Biclot et al., 2022; Bircher, Geirnaert, et al., 2018; Bircher, Schwab, et al., 2018; Chen, Hu, et al., 2022; Chen, Huo, et al., 2022; Chen, Li, et al., 2022; Fouhy et al., 2015; Li et al., 2023). However, there is still little knowledge regarding the type of protective matrices to be used to prevent cryoinjuries for various bacterial species that represent (typical) components of the human gut microbiota or for a mix of bacterial species forming an artificial gut microbiota that lacks the protective matrix naturally present in faecal samples.

Therefore, in this study, the effects that the most frequently used non‐penetrating cryoprotective agents may play in preserving the viability of 53 bacterial species, corresponding to representative human gut bacterial taxa, were first evaluated through a flow cytometry‐based bacterial cell viability assay after one and 3 months of cryopreservation at −80°C. Subsequently, the same cryoprotectants were employed to assess their ability to preserve the viability and taxonomic composition of 10 artificial gut microbiota assemblies obtained through a continuous fermentation model system with immobilized faecal microbiota from healthy donors. In the latter case, following 6 months of cryopreservation at −80°C, bacterial cell viability was evaluated through flow cytometry, while maintenance of the original biodiversity was assessed through shallow shotgun sequencing after revitalizing the cryopreserved artificial gut microbiota in an in vitro fermentation model.

## EXPERIMENTAL PROCEDURES

### Bacterial strains, culture conditions and storage

**FIGURE 1:**
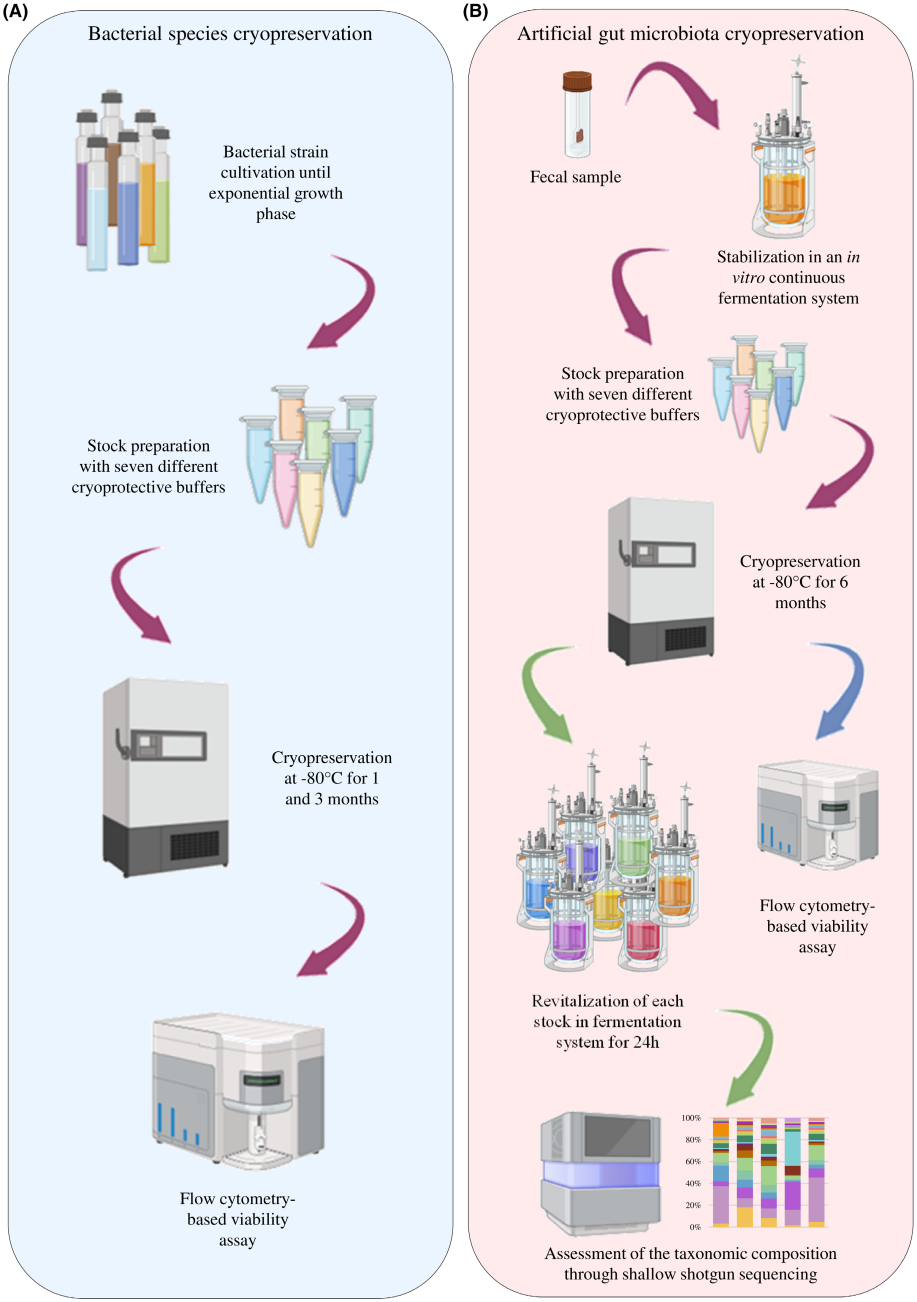
Cryopreservation workflow. Panel A depicts the schematic workflow used to cryopreserve bacterial strains representative of the human gut microbiota and the downstream analysis performed to assess bacterial cell viability after 1 and 3 months of cryopreservation at −80°C. Panel B shows the schematic procedure used to generate and cryopreserve artificial gut microbiota, as well as the analyses employed to assess artificial gut microbiota viability and biodiversity after 6 months of cryopreservation.

Fifty‐three bacterial species, belonging to the main taxa of the human gut microbiota (Tramontano et al., 2018; Zimmermann et al., 2019), were selected to assess the ability of various non‐penetrating cryoprotectants to preserve bacterial viability following exposure to −80°C. The selected bacterial strains, growth media and culture conditions are listed in Table S1. Specifically, for the cultivation of type strains, the growth medium recommended by the international strain repository from which they were purchased was used (Table S1), while bacterial strains isolated in the laboratory were cultivated in the growth medium used for their isolation (Table S1) (Alessandri et al., 2022; Huang et al., 2023). In the latter case, only bacterial strains isolated from human faecal samples were employed. All strains were revitalized from a glycerol stock (15% glycerol) and routinely cultivated in an anaerobic chamber (2.99% H_2_, 17.01% CO_2_ and 80% N_2_) at 37°C, using Hungate tubes for strictly anaerobic bacteria (Table S1). For each bacterial strain, after an overnight cultivation, 100 μL of the reactivated culture was transferred to 50 mL of a specific culture medium and incubated anaerobically at 37°C until an early stationary growth phase was reached (Bircher, Geirnaert, et al., 2018; Bircher, Schwab, et al., 2018). Subsequently, the bacterial culture was divided into 1 mL aliquots, and cells were harvested by centrifugation at 4°C for 8 min at 1000 *g*. Then, the obtained cell pellets were re‐suspended in 1 mL of each of the seven prepared cryoprotective buffers. After an incubation time of 30 min at room temperature to allow the cryoprotectant agents to interact with the bacterial cells, the mixtures of cryoprotectants and bacterial cells were stored at −80°C in screw‐cap polypropylene cryotubes (Sarstedt, Germany). A viability assay was conducted at three different time points: immediately before stock preparation (T0), as well as after one (T1) and three (T3) months of cryopreservation at −80°C. All procedures, except for centrifugation and the flow cytometry assay, were performed under anaerobic conditions. The workflow of this study is summarized in Figure 1.

### Cryoprotective solution preparation

Seven different cryoprotective buffers were prepared to store the selected bacterial strains at −80°C. All cryoprotective buffers were characterized by common components, i.e. peptone buffered water (final concentration of 0.1% w/v) and glycerol (final concentration of 15% v/v), which represent the most frequently used penetrating cryoprotective agents for their non‐toxic nature even at high concentrations (Bircher, Geirnaert, et al., 2018; Bircher, Schwab, et al., 2018); the reducing agents L‐cysteine‐HCl (1 g/L); and the co‐enzyme/vitamin riboflavin (0.3 g/L) to assist bacteria to protect themselves against oxidative damage/stress inflicted during processing and storage. This starting protective solution, here named Basal Solution (BS), was selected for its reported ability to preserve the viability of single bacterial strains and artificial gut microbiota (Bircher, Geirnaert, et al., 2018; Bircher, Schwab, et al., 2018) and also to avoid the supplementation of culture medium in the preparation of bacterial stocks that would introduce an additional experimental variable. The BS was then supplemented with other non‐penetrating cryoprotectants: (i) inulin (5% w/v), (ii) sucrose (5% w/v), (iii) skimmed milk (5% v/v), (iv) trehalose (5% w/v), (v) a mix of the three sugars with a final concentration of 5% w/v each and (vi) a mix of all tested cryoprotective agents. The percentage of sugars was selected based on previous studies (Bircher, Geirnaert, et al., 2018; Bircher, Schwab, et al., 2018; Hubalek, 2003; Smirnova et al., 2019). The BS solution was also included in the tested cryoprotective buffers as a control. The cryoprotective buffers, except for the skimmed milk, which was autoclaved at 121°C for 5 min and then added to the solutions, were filter‐sterilized by using filters with pores of 0.2 μm diameter, wrapped with aluminium foil to protect photosensitive agents and anaerobically incubated overnight to remove traces of oxygen until usage.

### Flow cytometry‐based cell viability assay

Upon thawing following 1 or 3 months of cryopreservation, an aliquot of each bacterial strain stock was evaluated for viability by means of flow cytometry. Specifically, the cryopreserved bacterial strains were diluted 100 times in Phosphate Buffered Solution (PBS) and the diluted cell suspension was then used for a flow cytometry‐mediated cell viability assay using the fluorescent dyes SYTO9 (3.34 mM) and Propidium Iodide (PI; 20 mM) of the LIVE/DEAD BacLight Bacterial Viability Kit (ThermoFisher Scientific, USA), following the manufacturer's instructions. Specifically, five different tubes of the diluted cells (final volume of 1 mL each) were obtained per thawed stock. In detail, one of the tubes was subjected to centrifugation at 3000 rpm for 8 min, the supernatant was discarded and the microbial cell pellet was resuspended in 70% isopropyl alcohol for 1 h to allow the permeabilization of the microbial cell membrane to induce cell death. Treated cells were then centrifuged, resuspended in PBS and dyed with the addition of 1.5 μL of PI. Two tubes were stained with 1.5 μL of one of the two dyes, while a fourth tube was not stained. Finally, the fifth tube was stained with both SYTO9 and PI. Immediately following staining, samples were vortex‐mixed and incubated in the dark for 15 min at room temperature. For instrument parameter adjustment, single‐dyed samples and the sample exposed to isopropyl alcohol were used as controls, while the non‐stained cells were used as a background control, as previously described (Lugli et al., 2021). A cell viability assay was performed with an Attune NxT flow cytometer (ThermoFisher Scientific, USA), and all data were analysed with the Attune NxT flow cytometry software. The viability assay was performed in triplicate.

### Ethics statement

The study protocol was approved by the Ethical Committee of the University of Parma, Italy. A signed informed consent was obtained from everyone enrolled in this study.

### Subject recruitment and sample collection

Fresh faecal samples were collected from ten human donors (average age of 32 years). To be enrolled, donors had to be healthy and not have undergone any treatment with prebiotics, probiotics or drugs during the 3 months prior to sample collection. Approximately 5 g of stool were collected immediately after defecation using a dedicated sterile tube provided by a sampling spoon containing 5 mL of sterile, pre‐reduced peptone water (0.1%, pH 7; Sigma Aldrich, Germany), as previously described (Bircher, Geirnaert, et al., 2018; Bircher, Schwab, et al., 2018). After collection, the faecal samples were immediately shipped to the laboratory under anaerobic conditions using a jar containing an anaerobic atmosphere generation bag (Thermo Scientific, USA).

### Faecal inoculum and immobilization

Once in the laboratory, tubes were transferred into an anaerobic workstation (2.99% H_2_, 17.01% CO_2_ and 80% N_2_), and pre‐reduced peptone water was added to the faecal sample to obtain a final concentration of 20% v/w (Bircher, Geirnaert, et al., 2018; Bircher, Schwab, et al., 2018). Subsequently, each faecal sample was immobilized in 1–2 mm gellan–xanthan gel beads (2.5% gellan gum, 0.25% xanthan gum and 0.2% sodium citrate), as previously described (Cinquin et al., 2004; Cleusix et al., 2008; Zihler Berner et al., 2013). The immobilization of the bacterial community of the faecal samples on gel beads was carried out to provide an anchoring substrate to the bacterial cells, favouring better preservation of the original taxonomic composition of the faecal sample over time. However, at the same time, since bacterial cell release occurs spontaneously from beads to the culture medium, the faecal immobilization on beads also allows to obtain a bacterial community representative of the original faecal sample directly in the culture medium, avoiding the collection of beads, as previously described (Champagne et al., 1994; Cinquin et al., 2004; Isenring et al., 2021; Zihler Berner et al., 2013).

### Bioreactor‐based in vitro cultivation of gut microbiota

To obtain artificial gut microbiota from the enrolled donors, a colonic fermentation system (Solaris Biotech Solutions, Italy) was set up and inoculated with immobilized gut microbiota. Specifically, 120 mL of freshly prepared faecal beads were transferred to 380 mL of sterile human colon environment‐simulating growth medium (Alessandri et al., 2022) supplemented with 1 mL/L vitamin (0.002 g/L d‐biotin, 0.01 g/L pantothenate, 0.005 g/L nicotinamide, 0.0005 g/L vitamin B12, 0.004 g/L thiamine, 0.005 g/L para‐aminobenzoic acid) and 1 mL/L mineral (0.5 g/L MnSO_4_ 2H_2_O, 0.1 g/L CoSO_4_, 0.1 g/L ZnSO_4_, 0.01 g/L CuSO_4_ 5H_2_O, 0.01 g/L AlKSO_4_, 0.01 g/L H_3_BO_3_, 0.01 g/L Na_2_MoO_4_ 6H_2_O, 0.1 NiCl_2_ 6H_2_O, 0.01 g/L Na_2_SeO_3_) solutions previously sterilized through a 0.2‐μm filter (Macfarlane et al., 1998). Furthermore, to mimic the intestinal environment, the cultivations were performed under anaerobic conditions through the flushing of an anaerobic mix (2.99% H_2_, 17.01% CO_2_ and 80% N_2_) in the fermentation system. The temperature was kept at 37°C with continuous stirring at 120 rpm, while the pH was maintained at 6.8 by the addition of 2.5 M NaOH (Macfarlane et al., 1998). To allow colonization of the beads, the bioreactor was first run as a closed system by replacing the exhausted medium every 12 h for a total fermentation time of 60 h. Subsequently, the cultivation was switched to a continuous mode by an inflow and outflow of fresh and exhausted growth medium, respectively, via peristaltic pumps with a mean retention time of 8 h (50 mL/h) mimicking the average colonic transit time in healthy adults (Isenring et al., 2022; Poeker et al., 2018). Stabilization of the gut microbiota was imposed following a 15‐day run of this model gut system in continuous mode, after which the microbial ecosystem was collected for cryopreservation (Bircher, Geirnaert, et al., 2018; Bircher, Schwab, et al., 2018).

### Cryopreservation of the artificial gut microbiota

For each stabilized gut microbiota, 150 mL of cultivation were directly collected from the bioreactor through a dedicated sterile sampling system (Solaris Biotech Solutions, Italy) by means of a sterile needle connected to a 50 mL syringe. Specifically, the latter was connected to a sampling tube equipped with a filter, which allows the suspension of cells and medium to be retrieved, avoiding the collection of gel beads. The latter were excluded from the collection to avoid introducing an experimental variable, as it would be impossible to introduce an equal number of beads per bacterial stock. The effluent was immediately transferred to a sterile, pre‐reduced tube and transported into an anaerobic chamber, where all further steps were performed. Specifically, effluent microbial biomasses were divided into sterile tubes containing 6 mL of the stabilized gut microbiota and harvested by centrifugation at 3000 rpm for 8 min. Subsequently, the supernatant of each tube was discarded, while the bacterial cell pellets were resuspended in 1 mL of each cryoprotective buffer to be tested (three bacterial stocks per cryoprotective solution were obtained). The obtained suspensions were then incubated at room temperature for 30 min to allow microbial cell‐protective agent interaction before transferring the obtained stocks at −80°C. The cryoprotective buffers were prepared following the same procedure used for the cryopreservation of single bacterial strains, as above described (Table S2). After 6 months of storage at −80°C, three stock samples per cryoprotective buffer for each stabilized gut microbiota were subjected to a flow cytometry‐based cell viability assay, following the same procedure as described above for single bacterial strains. Specifically, since the revitalization of AGMs for the evaluation of biodiversity conservation over time is highly time‐consuming, in the case of AGMs, contrary to individual bacterial species, the evaluation of viability and biodiversity was fixed after 6 months of cryopreservation.

### Revitalization of the artificial gut microbiota

In addition to bacterial cell viability assessment, after 6 months of cryopreservation at −80°C, a stock representing each cryoprotective buffer for each stabilized gut microbiota was also reactivated through a bioreactor‐based cultivation. The growth medium and the setup of the fermentation system were the same for gut microbiota stabilization, with the only exception of the fermentation time. Indeed, the cultivation was run for only 24 h with 12 h operating as batch cultivation and the other 12 h in a continuous mode. Each cryopreserved, stabilized gut microbiota was inoculated to reach a final concentration of 0.1% in a volume of 400 mL of growth medium. After 24 h cultivation, an aliquot of the bacterial culture was collected and stored at −20°C until DNA extraction. The entire workflow followed for viability and biodiversity assessment of artificial gut microbiota after cryopreservation at −80°C is summarized in Figure 1.

### DNA extraction and shallow shotgun sequencing

The 10 faecal samples and the corresponding in vitro stabilized gut microbiota, as well as the microbial community obtained after the revitalization of each stabilized gut microbiota per considered cryoprotectant, were subjected to microbial DNA extraction using the QIAmp DNA Stool Mini Kit (Qiagen, Germany), following the manufacturer's guidelines. The extracted DNA was prepared using the Illumina Nextera XT DNA Library Preparation Kit, following the Illumina Nextera XT protocol. In detail, DNA samples were enzymatically fragmented, barcoded and purified employing magnetic beads. Subsequently, samples were quantified using a fluorometric Qubit quantification system (Life Technologies, USA), loaded on a 2200 Tape Station Instrument (Agilent Technologies, USA) and normalized to 4 nM. Paired‐end sequencing was performed using an Illumina MiSeq sequencer (Illumina Inc., San Diego, USA) with the 2 × 250 MiSeq Reagent Kit v3 (600‐cycle) and a spike‐in of 1% PhiX control library.

### Analysis of shallow shotgun metagenomic datasets

The obtained raw data in .fastq format were filtered to remove reads with a quality of <25 and sequences corresponding to human DNA by mapping the reads on the *Homo sapiens* genome, while reads with a length of >149 bp were retained. Quality‐filtered data were used for further analysis with METAnnotatorX2 for taxonomic profile reconstruction, as previously reported (Milani et al., 2021). Retained sequences were used as input to perform a MegaBLAST local alignment of reads to a pre‐processed database, including available genomes of eukaryotes (Fungi and Protists), bacteria, archaea and viruses, following the METAnnotatorX2 manual (Milani et al., 2021). Reads showing a nucleotide identity of >94% to the genomes included in the database were classified at the species level, while if a lower percentage identity was detected, they were classified at the genus level as undefined species. Species richness represented the number of bacterial species detected for each metagenomic sample. Bray–Curtis dissimilarity matrices based on species abundance calculated through the Rstudios software were used to evaluate similarities between samples (β‐diversity). The similarity range was calculated as a value between 0 and 1. Principal coordinate analysis (PCoA) was used to represent β‐diversity using ORIGIN 2021 (https://www.originlab.com/2021). In the PCoA, each sphere represents a sample distributed in tridimensional space according to its specific taxonomic composition.

### Statistical analysis

SPSS software was used to compute statistical analysis. i.e., the ANOVA Bonferroni post‐hoc test and the Mann–Whitney *U* test.

### Data deposition

Raw sequences of shallow shotgun sequences are accessible through the sequence Read Archive (SRA) under the BioProject accession number PRJNA1084800.

## RESULTS AND DISCUSSION

### Impact of cryoprotectants on key members of the human gut microbiota

The availability in the laboratory microbial biobank of pure strains representing various bacterial species that commonly inhabit the human intestine is pivotal to performing in vitro investigations of microbe–microbe or microbe–host interactions, while such strains can also be used to generate synthetic gut microbiota mimicking the human gut microbial ecosystem (Aranda‐Diaz et al., 2022; Lagier et al., 2016, 2018). However, to ensure their survival and storage stability over time, bacterial strains require adequate and reliable conservation. In this context, cryopreservation at −80°C represents the most widely exploited protocol for long‐term bacterial storage as it avoids the mechanical, osmotic and oxidative stress to which bacteria are exposed when freeze‐drying is used as storage processing (Barzegari et al., 2014; Bircher, Geirnaert, et al., 2018; Bircher, Schwab, et al., 2018; Smirnova et al., 2019). However, since cryopreservation is also not exempt from causing lethal damage to bacterial cells, this storage method requires an accurate evaluation of the components with cryoprotective properties to be used to protect the bacterial biomass from ice formation (Biclot et al., 2022; Bircher, Geirnaert, et al., 2018; Bircher, Schwab, et al., 2018; Chen, Hu, et al., 2022; Chen, Huo, et al., 2022; Chen, Li, et al., 2022; Smirnova et al., 2019). In this context, different cryoprotective buffers were assayed for their ability to preserve the viability of 53 bacterial species representing common bacterial taxa of the human gut microbiota, as already pointed out in other studies that used bacterial species representing common taxa of the human gut microbiota (Table S1) (Tramontano et al., 2018; Zimmermann et al., 2019). Specifically, each bacterial strain was cultivated until the early exponential growth phase and subsequently stocked with seven different protective buffers. These buffers were characterized by a common formulation, including peptone buffered water, the non‐penetrating cryoprotectant glycerol and two reducing agents, i.e., L‐cysteine‐HCl and riboflavin, since these compounds have previously been described as efficient cryoprotectants in preserving the cell viability of both single bacterial strains and artificial gut microbiota (Bircher, Geirnaert, et al., 2018; Bircher, Schwab, et al., 2018; Khan et al., 2014). This formulation, here referred to as basal solution (BS), was then supplemented with non‐penetrating cryoprotective agents, resulting in the following buffers: (i) BS, (ii) BS + 5% inulin, (iii) BS + 5% skimmed milk, (iv) BS + 5% sucrose, (v) BS + 5% trehalose, (vi) BS + a mix of the abovementioned saccharides and (vii) BS + a combination of all tested non‐penetrating cryoprotective agents. In addition, for each bacterial strain, a flow cytometry‐based viability assay was performed at T0 (immediately before stock preparation), and after one (T1) and three (T3) months of cryopreservation at −80°C.

**FIGURE 2:**
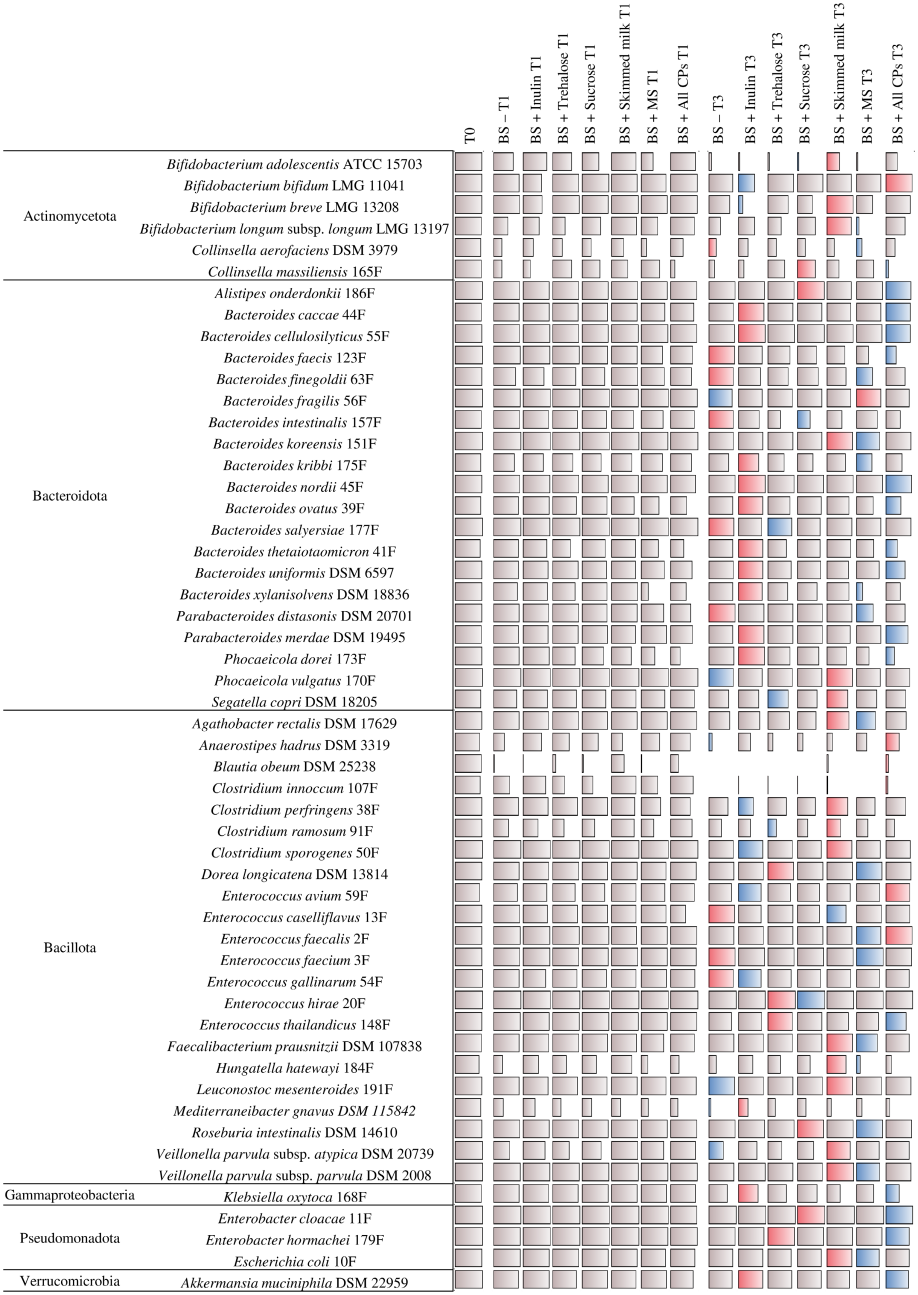
Bacterial cell viability after cryopreservation with different cryoprotective agents. The bar plot map reports the percentage of intact cells of 53 different bacterial species representative of the human gut microbiota immediately before cryopreservation (T0) and after one (T1) and three (T3) months of cryopreservation with seven different buffers. All data were obtained through a flow cytometry‐based assay and each percentage is expressed as the average of three replicates. The column on the left displays the bacterial phyla, while the central column reports the bacterial strains included in the study. MS, Mix of Sugars; CPs, Cryoprotective Agents. The highest and lowest percentage of viable cells for each bacterial species was highlighted with pink and light blue bar plots, respectively, at T3.

As expected, with a percentage of live cells ranging from 88.18% for *Hungatella hathewayi* to 99.98% for *Clostridium sporogenes* (average bacterial viability of 96.97%), the highest cell viability was recorded at T0 when compared to T1 and T3 for each bacterial strain (Figure 2 and Table S2). These findings confirm not only that almost all bacterial cells were viable at the time of storage at −80°C, but also that prolonged exposure to −80°C caused statistically significant reductions in cell viability for many of the tested strains (Figure 2 and Table S2). Indeed, while at T1, certain cryoprotectants seem to fully preserve the cell viability of many bacterial strains when compared to T0 (ANOVA Bonferroni post‐hoc *p*‐value > 0.05), at T3, a significant reduction in cell survival was observed for 47 out of the 53 selected bacterial strains with respect to T0 for all cryoprotectants (ANOVA Bonferroni post‐hoc *p*‐value < 0.05) (Figure 2 and Table S3). Nonetheless, the observed reduction of viable bacterial cells appeared to be cryoprotectant‐dependent for each bacterial strain (Figure 2, Tables S2 and S3), corroborating previous observations reported for certain strict anaerobic bacteria associated with the human gut (Bircher, Geirnaert, et al., 2018; Bircher, Schwab, et al., 2018).

Nevertheless, in‐depth assessment of viability revealed that the presence of skimmed milk not only allowed the highest level of viable cell recovery for the highest number of tested species (16 bacterial species at T3), yet, at the same time, it also induced a very high reduction in cell viability in only one species, i.e., *Enterococcus casseliflavus* (Figure 2 and Table S2). Thus, skimmed milk was selected as the best‐performing non‐penetrating cryoprotectant in terms of preserving the viability of bacterial cells when the latter were kept at −80°C for extended periods of time. In this context, the high protein content of skimmed milk may play a fundamental role in minimizing cellular cryoinjuries by stabilizing cell membrane components and providing a protective coating to bacterial cells, thus favouring preservation of cell integrity and viability (Bellali et al., 2020; Wang et al., 2019).

Next to skimmed milk, inulin was shown to be another high‐performing cryoprotectant, capable of preserving cell viability for 14 bacterial species at T3 (Figure 2 and Table S2). Not by chance, inulin‐type fructans have been described as powerful cryoprotective agents for their ability to directly interact with membrane lipids, stabilizing bacterial biomass under cold and dry conditions (Bircher, Geirnaert, et al., 2018; Bircher, Schwab, et al., 2018; Hubalek, 2003; Schwab et al., 2007). Therefore, preserving the highest percentage of viable cells for more than half of the tested bacteria (30 out of 53 bacterial species), inulin and skimmed milk can be considered as the best cryoprotectants among those tested to maintain cell viability in the case of cryopreservation of single bacterial strains. However, in contrast to skimmed milk, inulin was shown to be the worst cryoprotectant for six bacterial strains, including *Bifidobacterium breve*, *Clostridium perfringens* and *Enterococcus avium*, whose cell viability was better preserved using skimmed milk (Figure 2 and Table S2). These findings demonstrate that these two cryoprotectants are not interchangeable, but rather, that their cryopreservation ability is species‐specific. By dividing the tested strains by bacterial genus, when at least two members of the same genus were included in the study, cryoprotectant‐dependent viability patterns were identified by analysing the flow cytometry‐based viability data. Indeed, while inulin appeared to be the cryoprotectant that, in general, allowed high‐level retention of cell viability for bacterial species belonging to the genera *Bacteroides*, *Parabacteroides* and *Phocaeicola*, skimmed milk guaranteed a better recovery of intact cells for members of the genera *Bifidobacterium*, *Clostridium* and *Veillonella* (Figure 2 and Table S2).

Further detailed information about the ability of the other considered cryoprotective agents to preserve bacterial cell viability is reported in the supplementary text (Bircher, Geirnaert, et al., 2018; Bircher, Schwab, et al., 2018; Fowler & Toner, 2005; Kerckhof et al., 2014).

Therefore, overall, although the viability of bacterial strains strictly depends on the cryoprotective agent used, these data not only suggest skimmed milk and inulin as cryoprotectants to be preferred to preserve cell viability of bacterial species typical of the human gut microbiota, but also that the addition of a single non‐penetrating cryoprotectant guarantees superior cryopreservation of cell viability for single bacterial strain cryopreservation when compared to the use of multiple combined cryoprotective agents.

### Taxonomic insight into the artificial gut microbiota

In recent years, in vitro stabilization of the human intestinal microbial community by means of continuous fermentation models has become an increasingly exploited technology to generate artificial gut microbiota (Deschamps et al., 2020; Isenring et al., 2023; Singh et al., 2022). In this scenario, beyond the cryopreservation of individual strains, the need to identify which cryoprotectants are most suitable not only for preserving the viability, but also the biodiversity of in vitro‐generated artificial gut microbiota, has become increasingly demanding. Therefore, to identify the best performing cryoprotectant for the preservation of cell viability and biodiversity of human artificial gut microbiotas (AGMs), ten faecal samples collected from healthy adults were separately inoculated as bead‐immobilized faecal microbial communities in continuous cultivation systems, consisting of a single bioreactor simulating the human colon environment, for 15 days. This growth period was selected as the average time required to stabilize a human faecal microbial community in an in vitro continuous cultivation system, as demonstrated in previous studies (Asare et al., 2023; Pham et al., 2019). At the end of the cultivation period, an aliquot of each artificial gut microbiota, as well as the original faecal samples, were subjected to species‐level taxonomic profiling through shallow shotgun sequencing to check whether the stabilized gut microbiota was representative of the corresponding inoculated faecal samples. The sequencing generated a total of 1,781,190 reads, resulting in 1,046,641 reads with an average of 52,332 reads per sample after quality filtering (Table S4).

**FIGURE 3:**
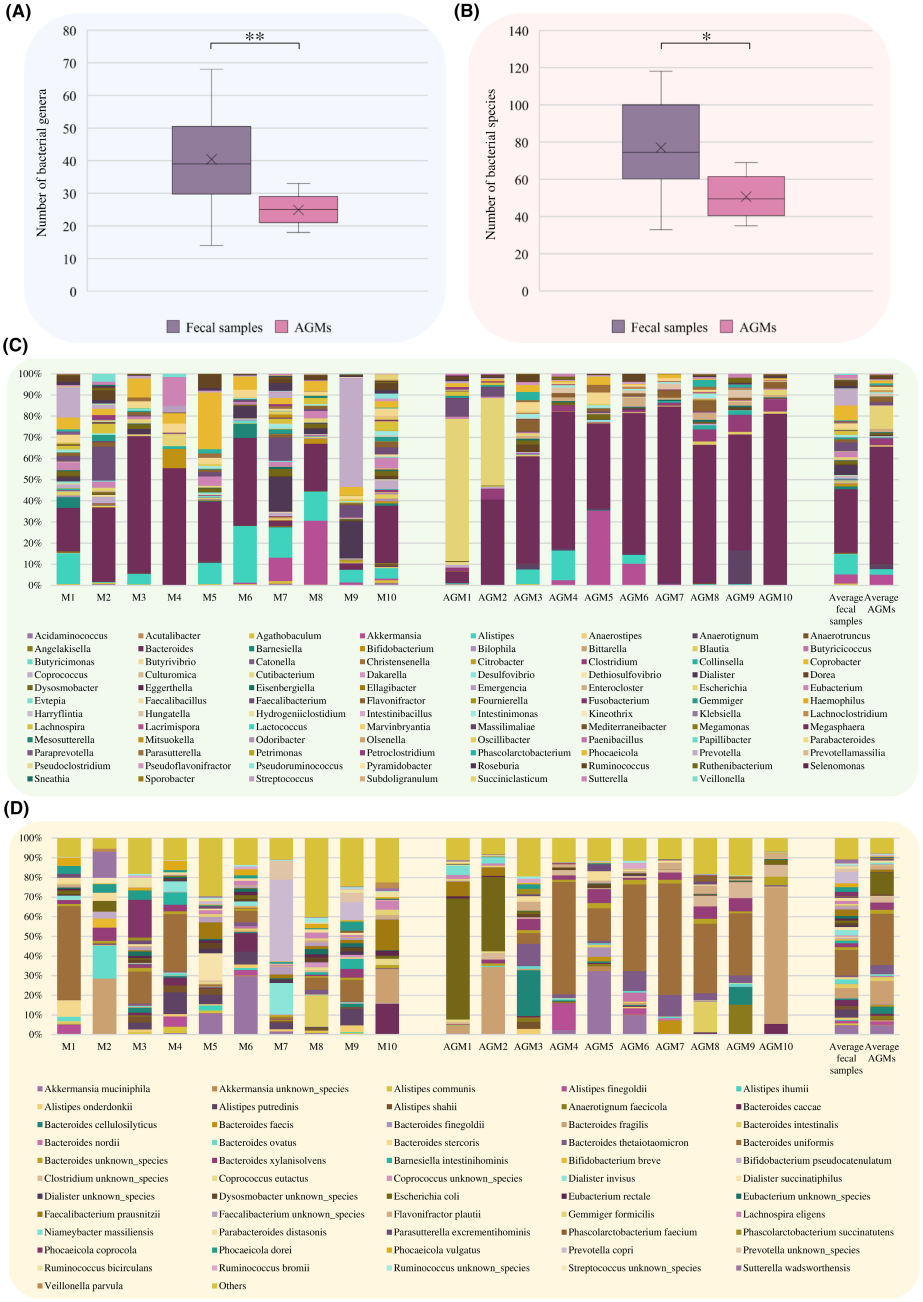
Taxonomy‐based comparison between the collected faecal samples and the corresponding artificial gut microbiota obtained through continuous fermentation systems. Panels A and B show the box and whisker plots of the calculated average number of bacterial genera and species, respectively, detected in the faecal samples and in the corresponding AGMs. The x‐axis reports the two considered groups, while the y‐axis shows the number of bacterial genera and species in panels A and B, respectively. Boxes are determined by the 25th and 75th percentiles. The whiskers are determined by the maximum and minimum values and correspond to the extreme values in the box. Lines inside the boxes represent the average, while crosses correspond to the median. Panels C and D illustrate the genus‐ and species‐level taxonomic composition, respectively, of the collected faecal samples and the corresponding AGMs.

As expected, analysis of the shallow shotgun sequencing data revealed a reduced microbial biodiversity of the artificial gut microbiota when compared to the faecal samples. Specifically, a significant reduction in the number of detected bacterial genera was recorded in the AGMs when compared to the faecal samples (Mann–Whitney *U* test *p*‐value = 0.006), with an average of 25 and 40 bacterial genera identified in the AGMs and original faecal samples, respectively (Figure 3A and Table S5). A decrease was also observed at the species level. Indeed, the species richness analysis highlighted a significant reduction in the number of distinct bacterial species identified in the AGMs when compared to those observed in the corresponding faecal samples, with an average number of bacterial species of 50 and 77, respectively (Mann–Whitney *U* test *p*‐value = 0.013) (Figure 3B and Table S6). This indicates that the obtained AGMs, even if cultivated in a human gut‐simulating environment, underwent an inevitable simplification of their bacterial community, resulting in reduced microbial biodiversity, as previously observed (Bircher, Geirnaert, et al., 2018; Bircher, Schwab, et al., 2018; Fehlbaum et al., 2015; Rachmuhl et al., 2023). Indeed, while *Akkermansia*, *Alistipes*, *Bacteroides*, *Dialister*, *Faecalibacterium*, *Phocaeicola*, *Prevotella* and *Sutterella* corresponded to the most representative bacterial genera of the collected faecal samples (relative abundance >10% in at least one sample) (Figure 3C and Table S5), only *Akkermansia*, *Alistipes* and *Bacteroides* maintained high relative abundances (>10%) in the AGMs (Figure 3C and Table S5). In this context, except for AGM1 and AGM2, for which a dominance of *Escherichia coli* was recorded, in all other stabilized faecal samples a conspicuous increment of members of the genus *Bacteroides* at the expense of other bacterial species was observed (Figure 3C, Tables S5 and S6), with *Bacteroides cellulosilyticus*, *Bacteroides fragilis*, *Bacteroides intestinalis*, *Bacteroides thetaiotaomicron*, *Bacteroides uniformis* and *Bacteroides xylanisolvens* as the most dominant *Bacteroides* species of the AGMs. However, in depth insights into the taxonomic composition of both faecal samples and in vitro cultivated microbial community revealed that, in addition to *Bacteroides*, other main players of the human gut microbiota were stabilized in the AGMs, albeit at lower relative abundances, including *Akkermansia muciniphila*, *Alistipes onderdonkii*, *Anaerotignum faecicola*, *Collinsella aerofaciens*, *Dialister succinatiphilus*, *Dorea longicatena*, *Faecalibacterium prausnitzii*, *Parabacteroides distasonis*, *Parabacteroides merdae*, *Phocaeicola dorei*, *Phocaeicola vulgatus*, *Roseburia intestinalis* and *Mediterraneibacter gnavus* (a recent reclassification of *Ruminococcus gnavus*) (Togo et al., 2018) (Figure 3D and Table S6). The only exception was represented by *Segatella copri* (recent reclassification of *Prevotella copri*) (Hitch et al., 2022) which, although with a relative abundance of 41.70% and 9.19% in faecal samples M7 and M9, respectively, was not stabilized in the corresponding AGMs (Figure 3D). Probably, its low tolerance to oxygen and its peculiar nutritional requirements make the cultivation and stabilization of *Segatella copri* particularly difficult in in vitro fermentation systems (Chen, Hu, et al., 2022; Chen, Huo, et al., 2022; Chen, Li, et al., 2022; Huang et al., 2021).

However, despite the reduction of the relative abundance of certain bacterial players and the lack of stabilization of some abundant bacterial taxa typical of the human gut microbiota, including *Segatella copri*, the applied fermentation protocol allowed the retention and stabilization of both dominant and accessory bacterial species that characterize the collected faecal samples. In addition, all AGMs were characterized by high relative abundances of members of the genus *Bacteroides*, known to be one of the most abundant and prevalent bacterial genera of the human gut microbiota (Arumugam et al., 2011; Costea et al., 2018; Mancabelli et al., 2017). Thus, overall, these results strengthen the effectiveness of the fermentation protocol in sustaining the growth of the human gut microbiota, providing AGMs that could be considered as a valid representation of the original faecal samples.

### Effect of different cryoprotective agents on the viability of bacterial cells that make up the artificial gut microbiota

Once the faecal samples inoculated in the continuous fermentation system were stabilized, the bacterial biomasses were recovered, resuspended in the seven different protective buffers above used for the cryopreservation of single bacterial strains, and stored at −80°C. In addition, an aliquot of the recovered cells was subjected to a bacterial cell viability assay through flow cytometry to assess the vitality of bacteria before cryopreservation (T0). Furthermore, after 6 months of cryoexposure, to assess the efficacy of cryoprotectants to preserve the viability of the stabilized gut microbiota, the solutions were thawed and immediately subjected to a bacterial cell viability assay (T6).

**FIGURE 4:**
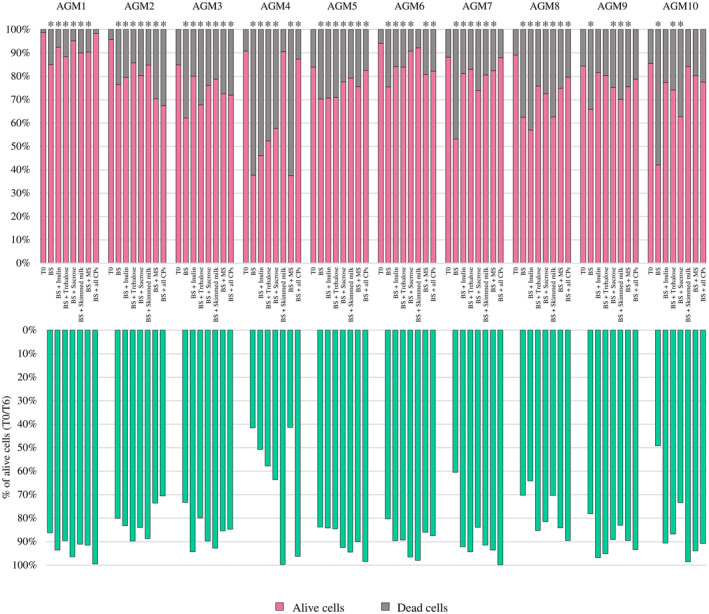
Flow cytometry‐based cell viability assessment of the AGMs after 6 months of cryopreservation. The bar plots at the top show the percentage of intact and dead bacterial cells immediately before storage at −80°C (T0) and after 6 months of cryopreservation for each artificial gut microbiota per cryoprotective agent, while the bar plots at the bottom indicate the percentage of survival rate for each AGM per cryoprotective agent when compared to T0. *p‐value < 0.05.

Interestingly, the survival rate of the bacterial cells constituting the various stabilized gut microbiota ranged from a minimum of 41.35% to a maximum of 99.82% when comparing the cell viability results from T6 to those from T0 (Figure 4). In detail, for all assessed stabilized gut microbiota, a significant reduction in cell viability was observed when the AGMs were cryopreserved with BS (ANOVA Bonferroni post‐hoc *p*‐value < 0.05) (Table S7). Effectively, BS was shown to be the cryoprotective solution that allowed the lowest level of survival of cryopreserved bacterial cells at T6 for all cases, except for AGM2 (Figure 4 and Table S7). This suggests that, beyond the presence of the penetrating cryoprotectant glycerol, the inclusion in the formulation of protective buffers of one or more non‐penetrating cryoprotective agents is required to ensure appropriate survival levels of bacterial cells in an artificial gut microbiota over time. Therefore, unlike what was observed for single bacterial strains for which BS ensured appreciable bacterial cell viability, it seems that cryopreservation of multiple species simultaneously requires the presence of, at least, a non‐penetrating cryoprotectant to better preserve bacterial cell viability.

Furthermore, as observed for the cryopreservation of single bacterial strains, in‐depth insight into the flow cytometry‐based viability data showed that there is not a single cryoprotective solution that guarantees optimal bacterial cell survival for each stabilized gut microbiota (Figure 4 and Table S7). In contrast, our results suggest that the ability of a single cryoprotective agent to preserve the viability of bacterial cells strictly depends on the (composition of the) stabilized microbial community (Figure 4 and Table S7). However, with a non‐significant reduction in cell viability in five out of 10 cases, i.e., AGM1, AGM5, AGM7, AGM9 and AGM10 (ANOVA Bonferroni post‐hoc *p*‐value > 0.05), and the lowest reduction in cell viability, even if statistically significant, recorded for AGM8 at T6 when compared to T0, the protective buffer containing all the tested cryoprotectants appeared to be the solution that, in general, allowed superior preservation of bacterial viability in AGMs (Figure 4 and Table S7). Probably, unlike what was observed for cryopreservation of single bacterial strains, the concerted action of multiple non‐penetrating cryoprotectants is necessary to better preserve the viability of the bacterial species present in the AGMs. This indicates that the different bacterial species included in the AGMs require different non‐penetrating cryoprotectants to be best preserved, and, therefore, only a mix of all cryoprotectants ensures optimal recovery of bacterial cells.

However, even the addition of skimmed milk alone to BS appeared to have an appreciable cryoprotective effect on AGMs. Indeed, not only for AGM4, AGM6 and AGM10, this cryoprotectant induced a non‐significant reduction in cell viability at T6 with respect to T0 (ANOVA Bonferroni post‐hoc *p*‐value > 0.05), but it also allowed bacterial survival of >90% for 7 AGMs after 6 months of cryoexposure (Figure 4 and Table S7). Similarly, guaranteeing a non‐significant reduction in cell viability for two AGMs (ANOVA Bonferroni post‐hoc *p*‐value > 0.05), i.e., AGM9 and AGM10, as well as the highest recovery in cell viability for AGM3, inulin can also be considered as an excellent cryoprotectant to preserve the viability of the several bacterial species composing the AGMs (Figure 4). Not by chance, this cryoprotective agent also allowed a survival rate higher than 90% from T0 to T6 for half of the AGMs (Figure 4).

Overall, these results extend, from single bacterial cells to complex bacterial communities, the notion that the ability of cryoprotective agents to preserve bacterial viability strictly depends on the specific microbial community to be cryopreserved. However, this data also highlights that, in the case of complex microbial communities, BS alone is not sufficient to guarantee bacterial viability. On the contrary, it seems that, to recover a higher number of viable bacterial cells from the AGMs, the synergistic action of all tested cryoprotectants is necessary. Indeed, although for individual microbial species it seemed that the combination of different non‐penetrating cryoprotectants triggers a competition between cryoprotective agents, inducing a lower preservation of bacterial cell viability, in complex microbial communities, the simultaneous availability of different bacterial species for which the cryoprotectants have differential affinities may contribute to decrease in the level of competition among cryoprotectants, resulting, rather, in synergistic activities.

However, as also observed for single bacterial strains, BS supplemented with skimmed milk or inulin may be considered as an excellent cryoprotective buffer to preserve AGM cell viability. In this context, since most of the AGMs were characterized/dominated by those species that resulted in being better cryopreserved with these two non‐penetrating cryoprotective agents when cryopreserved as single species, including *Bacteroides* species, it can be suggested that inulin and skimmed milk play a crucial role in preserving AGMs due to their high affinity for the species composing the AGMs.

Preservation of the viability of AGM bacterial cells is not the only prerequisite for evaluating the effectiveness of cryoprotective agents. Indeed, it is also necessary that cryoprotectants ensure the maintenance of the biodiversity of the original artificial gut microbiota.

### Cryoprotective agents impact the biodiversity of the artificial gut microbiota

To investigate the ability of each cryoprotectant to preserve the biodiversity of the obtained artificial gut microbiota, upon thawing of the latter to evaluate cell viability after 6 months of cryopreservation, an aliquot of each stock was also used as an inoculant in a bioreactor system. Specifically, the revitalization of the bacterial cells was carried out by setting the same parameters used for gut microbiota stabilization, yet without the addition of beads and for a total cultivation time of 24 h, running as a closed system for the first 12 h and in continuous mode for the remaining time. At the end of the cultivation, the obtained bacterial cultures were subjected to microbial profiling, resulting in a total of 7,961,081 reads, reduced to 4,366,043 reads with an average of 62,372 reads per sample after quality‐filtering (Table S8).

**FIGURE 5:**
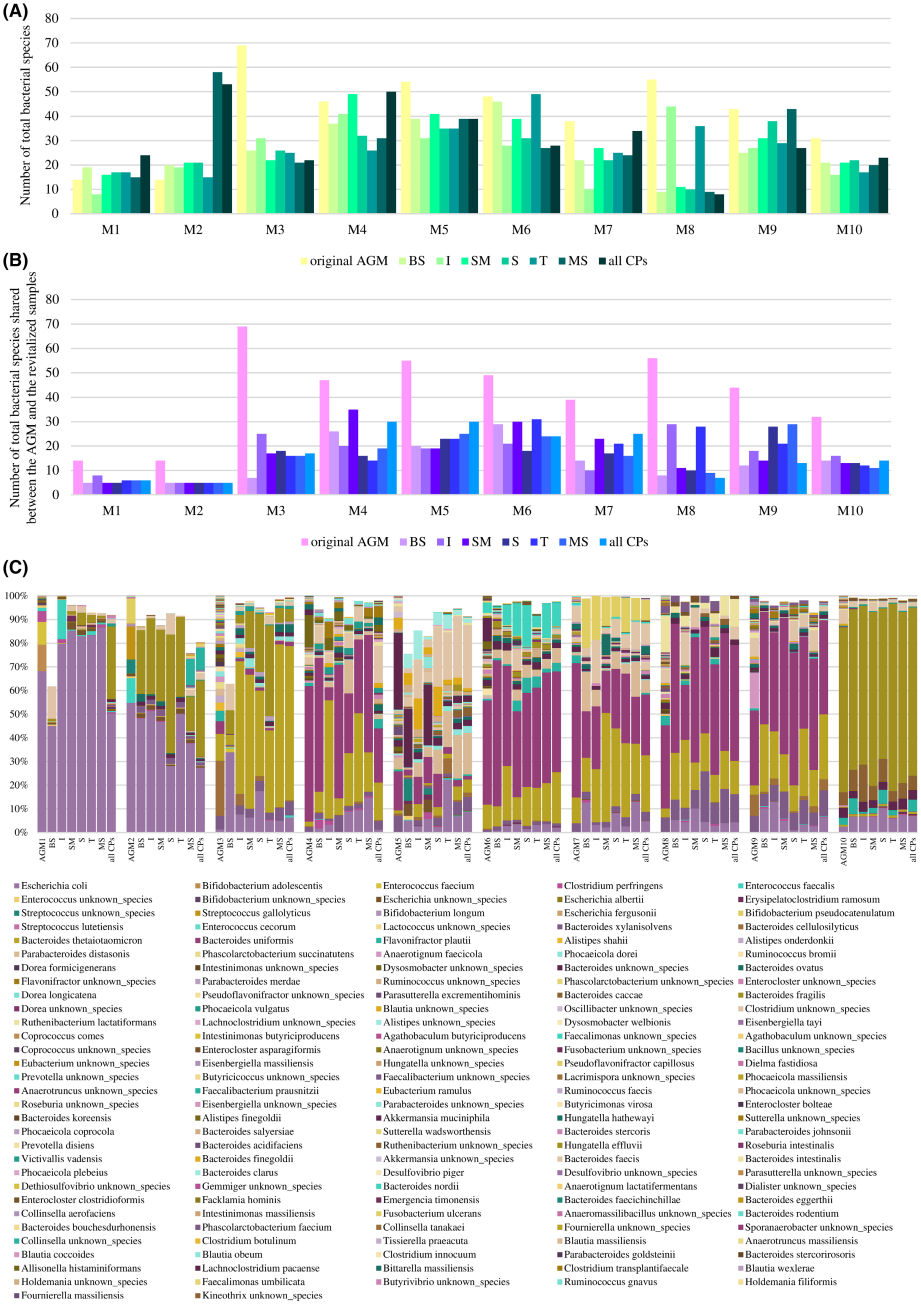
Effect of cryoprotectants on AGM cell viability and microbial biodiversity. Panel A reports the number of bacterial species detected in each AGM before storage at −80°C (original AGM) and after AGM revitalization after 6 months of cryopreservation for each cryoprotective agent. Panel B shows the number of bacterial species shared between the original AGMs and the revitalized AGMs per cryoprotective agent. Panel C depicts the relative abundance of the bacterial species detected in the AGMs. Only those bacterial species shared with the original AGMs are reported in the bar plots corresponding to the revitalized AGMs. MS, mix of sugars; CPs, cryoprotective agents.

Surprisingly, the number of species detected in the revitalized microbial communities after cryopreservation in different cryoprotective buffers was not always lower, as expected, than the number of species observed in the original AGMs (Figure 5A). Probably, the intrinsic depth bias of metagenomics approaches, whereby certain low‐abundant species in AGMs, even if present, may not be detected, led to an alteration of the real number of species (Alessandri et al., 2020; Amrane et al., 2018; Lagier et al., 2018). Alternately, cryopreservation could have induced the devitalization of a portion of cells of some more abundant bacterial species, allowing, in the case of revitalization, minor species to better compete in the human intestine‐simulating environment, favouring their growth and, therefore, their metagenomic detection.

Beyond the number of detected species, to preserve the stabilized bacterial community as well as its metabolic functions, it is essential that cryoprotectants preserve the highest number of bacterial species in the original AGMs as well as their initial relative abundances. In this context, as also observed for AGM bacterial cell viability, the BS alone or the BS supplemented with sucrose never allowed the highest number of species of the original AGM to be recovered in the revitalized samples (Figure 5B), indicating that other formulations of cryoprotective buffers are to be preferred not only to best preserve the bacterial viability but also to recover a higher number of bacterial species from the AGMs.

Conversely, inulin allowed the recovery of the highest number of bacterial species of the original AGM taxonomic composition for AGM1, AGM3, AGM8 and AGM10, while the supplement of skimmed milk, trehalose and a mix of saccharides to the BS allowed the revitalization of the highest number of the original AGM microbial taxa for AGM4, AGM6 and AGM9, respectively (Figure 5B). On the other side, the mixture of all cryoprotectants favoured the best conservation of the bacterial species from the cryopreserved AGM5 and AGM7 (Figure 5B), while for AGM2, no differences were observed among cryoprotectant agents in their ability to ensure the survival of the highest number of bacterial species from the original AGM. Indeed, all seven tested cryoprotective buffers were able to revitalize five species from the original AGM2 (Figure 5B). In this context, as already observed through the viability assay, this data underlines the impossibility of identifying a universal non‐penetrating cryoprotective agent able to guarantee recovery of all original AGM bacterial species following revitalization in a bioreactor system. This, again, emphasizes that the performance of various cryoprotectants in preserving bacterial species from cryoinjuries strictly depends on the particular taxonomic composition of the original AGM.

Furthermore, to identify the best‐performing cryoprotectant for maintaining biodiversity in the AGMs, a Bray–Curtis dissimilarity‐based β‐diversity analysis, represented through a principal coordinate analysis (PCoA), was performed for each AGM together with the corresponding revitalized samples stored at −80°C with each cryoprotectant (Figure 5C, Figure S1 and Table S9). This analysis highlighted a marked separation of the revitalized samples from the corresponding AGM, thus suggesting that cryoexposure, despite the presence of cryoprotective agents, induced an inevitable alteration of the AGM biodiversity (Figure S1). Furthermore, the β‐diversity analysis showed that a single cryoprotectant acts with different efficiency in preserving the bacterial biodiversity among the stabilized AGMs, thus indicating that, not only for cell viability and the number of species, but also for the maintenance of the biodiversity, it is not possible to identify a single cryoprotectant that is optimal for the cryopreservation of stabilized AGMs regardless of their taxonomic composition (Figure S1). In this context, based on the values generated through the calculation of the Bray–Curtis dissimilarity matrix, inulin seemed to better preserve the biodiversity of AGM1, AGM2 and AGM3, while for AGM4 and AGM10, the biodiversity was better conserved with the addition of skimmed milk to the BS (Figure 5C, Tables S9 and S10). In contrast, sucrose allowed a better recovery of bacterial biodiversity for AGM5 and AGM9; trehalose for AGM6 and AGM8; and the mixture of all cryoprotectants for AGM7 (Figure 5C, Tables S9 and S10). Probably, the different non‐penetrating cryoprotective agents interact differently with the cell envelope of various bacterial species present in the AGMs, leading to different performances in preserving them both in terms of cell viability and biodiversity (Bircher, Geirnaert, et al., 2018; Bircher, Schwab, et al., 2018; Leslie et al., 1995; Smirnova et al., 2019). Indeed, especially the non‐penetrating saccharides, anchoring to the bacterial cell membranes, may act as an easily accessible growth source to sustain the increased nutritional requirement of cryo‐stressed bacterial cells immediately after revitalization (Bircher, Geirnaert, et al., 2018; Bircher, Schwab, et al., 2018; Smirnova et al., 2019). This may explain, how in some cases, certain bacterial species showed higher relative abundances in the revitalized samples cryopreserved with carbohydrates as cryoprotectants when compared to the original AGMs.

Based on these results, a combination of the viability data with that associated with the maintenance of a higher number of AGM species and their relative abundances may be necessary to better understand the impact of each cryoprotectant on AGMs.

### Identification of the most effective cryoprotectants to store AGMs over time

Since a single cryoprotective agent able to guarantee both the preservation of the highest number of viable cells and the recovery of the original AGM biodiversity was not identified, data obtained from the viability assay and shallow shotgun sequencing of the revitalized samples were analysed in combination. In this context, for AGM3, AGM4, AGM7 and AGM10, the cryoprotectants that allowed the highest levels of viable cells to be recovered after 6 months of cryopreservation corresponded to the ones that also allowed the revitalization of the highest number of bacterial species from the original AGM and, therefore, to better conserve AGM biodiversity, i.e., inulin (for AGM3), skimmed milk (for AGM4 and AGM10) and all cryoprotectants (for AGM7) (Figures 4 and 5). For all other AGMs, on the contrary, no correlation was identified between the most effective cryoprotectants in maintaining cell viability and that preserving the highest number of bacterial species and their relative abundances. However, despite this apparent discrepancy, in‐depth scrutiny of the obtained results revealed that for AGM1, AGM2, AGM5, AGM6, AGM8 and AGM10, three cryoprotective buffers, i.e., inulin, skimmed milk or all tested cryoprotectants, allowed the retention of a biodiversity similar to that of the original AGMs as well as a high percentage of bacterial cell viability (Figures 4 and 5). This finding shows that, even if they did not provide the best results in terms of recovery of microbial biodiversity and/or cellular viability of AGMs, BS supplemented with inulin, skimmed milk or all tested cryoprotectants (probably for the synergistic action of inulin and skimmed milk) is to be preferred to other formulations for optimal cryopreservation of stabilized gut microbial communities regardless of their taxonomic composition.

Overall, the combination of viability and shallow shotgun results after revitalization of cryopreserved AGMs emphasizes that the inclusion of one or more non‐penetrating cryoprotectant(s) in the BS induced better preservation of cell viability and microbial biodiversity of the AGMs. However, differences in preserving AGM bacterial cell viability and microbial biodiversity were observed among the various non‐penetrating cryoprotectants, indicating that the tested cryoprotective agents cannot be supplemented with the BS indiscriminately to obtain a high level of preservation. Indeed, the obtained results highlighted that only the addition of inulin, skimmed milk or a combination of the tested cryoprotectants provides better recovery of both bacterial cell viability and AGM biodiversity, thus confirming that the combination of cryoprotective buffers is not only ideal for preserving the viability of single bacterial strains but also for complex microbial communities as AGMs.

## CONCLUSIONS

The availability of bacterial biobanks representative of the human gut microbiota and/or artificial gut microbiota communities is of fundamental importance for investigating the molecular mechanisms that regulate microbe–microbe and microbe–host interactions, avoiding the ethical concerns of in vivo studies. However, to preserve the viability of bacterial strains or artificial gut bacterial communities over time, adequate maintenance protocols for microbial isolates are necessary. In this study, the comparison of the survival rates of individual bacterial species representative of the human intestinal microbiota after cryopreservation at −80°C with different non‐penetrating cryoprotective agents revealed that the effectiveness of various tested cryoprotectants is species‐dependent. However, although a universal cryoprotectant capable of guaranteeing the highest recovery of intact bacterial cells was not identified, the inclusion of inulin or skimmed milk in the BS provided a higher level of protection during cryopreservation of single bacterial cells. Similar results were obtained even in the case of cryoconservation of complex microbial communities. Indeed, the cryopreservation of artificial gut microbial communities obtained by stabilizing faecal samples in continuous fermentation systems showed that it was not possible to identify a cryoprotective agent that simultaneously guaranteed the recovery of viable cells and the conservation of the initial biodiversity characterizing the artificial gut communities for all the AGMs. However, in this case, the combination of data concerning cell viability and microbial biodiversity highlighted that the supplementation of inulin, skimmed milk or all tested cryoprotectants with glycerol and non‐reducing agents allowed for higher protection of both viability and biodiversity of compositionally different artificial gut microbiota during cryopreservation.

Therefore, overall, these data emphasize the need to combine both penetrating (glycerol) and non‐penetrating cryoprotective agents to ensure improved cell survival in the case of cryopreservation of both single bacterial cells and complex microbial communities. In addition, this finding suggests that, despite the impossibility of electing a universal cryoprotectant, inulin and skimmed milk are to be considered non‐penetrating cryoprotectants that perform better in preserving cell viability and microbial biodiversity than others widely used in cryopreservation, such as sucrose or trehalose. Furthermore, the obtained data clearly show a reduction in viability as the cryopreservation time increases for both individual strains and artificial gut microbial communities, suggesting that, to ensure constant viability of bacterial strains/AGMs, it would be appropriate to replace bacterial stocks with fresh ones frequently. However, studies that take into consideration the assessment of viability for multiple time points and for a longer period are necessary to provide precise and detailed indications on the optimal times to obtain new stocks and guarantee bacterial survival. However, since the obtained data are exclusively based on viability assays via flow cytometry, viability data obtained through plate counting are required to corroborate flow cytometry‐based data, thus providing more robust and reliable results. At the same time, although the focus of this study is to evaluate how different cryoprotectants preserve the viability and biodiversity of different AGMs to better maintain the different taxonomic combinations over time, an evaluation of the effect that the selected cryoprotectants exert directly on the faecal samples could help identify the better performing cryoprotectants more robustly and for a wide range of sample types. In addition, the evaluation of the biodiversity that is recovered, compared to the original AGM sample, from the combination of two or more stocks prepared with different cryoprotectants could provide more information on how to maximize the recovery of AGM biodiversity after revitalization. Finally, although the amount of sugar added to the cryoprotectant solutions was chosen on the basis of previous studies, the evaluation of the impact that different concentrations of carbohydrates may have on the preservation of bacterial cell viability could be useful to define detailed guidelines for the preparation of bacterial stocks.

## AUTHOR CONTRIBUTIONS

**Giulia Alessandri:** Writing – original draft; investigation; conceptualization; formal analysis; data curation; visualization; validation; methodology. **Sonia Mirjam Rizzo:** Investigation; methodology; data curation; formal analysis. **Leonardo Mancabelli:** Conceptualization; investigation; visualization; validation; methodology; software; formal analysis; data curation. **Federico Fontana:** Software; formal analysis; data curation; methodology. **Giulia Longhi:** Investigation; methodology; formal analysis. **Francesca Turroni:** Writing – review and editing; resources; supervision. **Douwe van Sinderen:** Writing – review and editing; resources; supervision. **Marco Ventura:** Conceptualization; writing – review and editing; supervision; resources.

## FUNDING INFORMATION

F.F. and G.L. postdoctoral fellowship was funded by the National Recovery and Resilience Plan (NRRP), Mission 4 Component 2 Investment. 1.3 – Call for tender No. 341 of 15 March 2022 of the Italian Ministry of University and Research, funded by the European Union – NextGenerationEU; Award Number: Project code PE00000003, Concession Decree No. 1550 of 11 October 2022, adopted by the PRIN, Italian Ministry of University and Research, and European Union, Project title “ON Foods – Research and innovation network on food and nutrition Sustainability, Safety and Security – Working ON Foods”. Part of the Figure S1 were generated with BioRender.com.

## CONFLICT OF INTEREST STATEMENT

The authors declare no competing interests.

## Supporting information


Figure S1

**Tables S1** **–S10**

## Data Availability

The data that support the findings of this study are openly available in NCBI SRA at https://www.ncbi.nlm.nih.gov/sra, reference number PRJNA1084800.
