# Supplementary figures and images for: Impact of cryoprotective agents on human gut microbes and in vitro stabilized artificial gut microbiota communities
Source: Microb Biotechnol. 2024 Jun 15;17(6):e14509. doi: 10.1111/1751-7915.14509 (PMC11179620; doi:10.1111/1751-7915.14509)

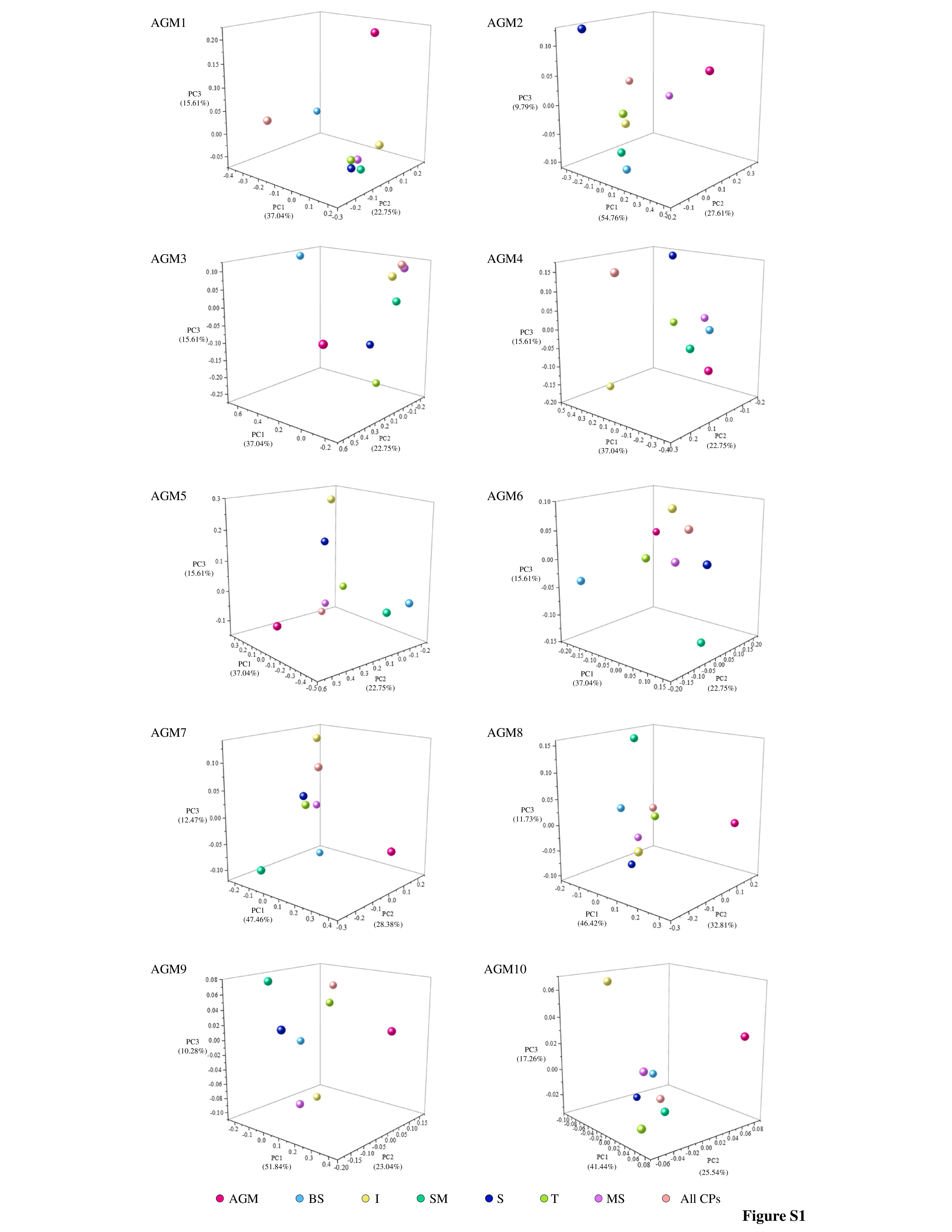

Supplement: Supplementary file 1 — Figure S1 [file MBT2-17-e14509-s002.tif]
